# Extremely Prolonged Ventricular Asystole

**Published:** 2009-09-01

**Authors:** Miguel A Arias, Alberto Puchol, Eduardo Castellanos, Marta Pachon, Luis Rodriguez-Padial

**Affiliations:** Cardiac Arrhythmia and Electrophysiology Unit. Department of Cardiology. Hospital Virgen de la Salud. Toledo. Spain

**Keywords:** prolonged asystole

## Abstract

We are reporting an extremely prolonged sinus arrest documented by Holter monitoring.

A 77-year-old woman was admitted to the geriatric unit at our institution. She complained of recurrent episodes of a sudden loss of consciousness at rest followed by 'seizures' lasting several minutes with complete spontaneous recovery after a few seconds. The patient had suffered multiple episodes in the previous weeks. She had a moderate degree of senile dementia and was not taken any medication. A blood analysis was unremarkable and the ECG showed normal sinus rhythm at 60 bpm with normal PR interval and QRS length. An echocardiogram revelead no significant cardiac structural abnormalities. Twenty-four hours ECG Holter recording was performed and the patient presented a syncopal episode while she was sitting on the armchair. Patient recovered completely after the episode. Holter examination revealed predominant sinus bradycardia (mean of 50 bpm) with no significant atrial or ventricular arrhythmia. At 19:34 pm, when she presented the syncopal attack, a gradual slowing of the sinus beats was observed followed by a sudden drop of ventricular activity due to extremely prolonged sinus arrest (2 minutes and 18 seconds) with two escape beats in between. The episode was terminated by 4 idioventricular beats followed by an accelerated junctional rhythm. Although a malignant vasovagal syncope can not be ruled out, lack of significant heart rate acceleration before ventricular asystole was in favour of sinus node dysfunction. A permanent pacemaker was implanted and no recurrences of syncopal episodes occurred during a year follow-up.

## Figures and Tables

**Figure 1:**
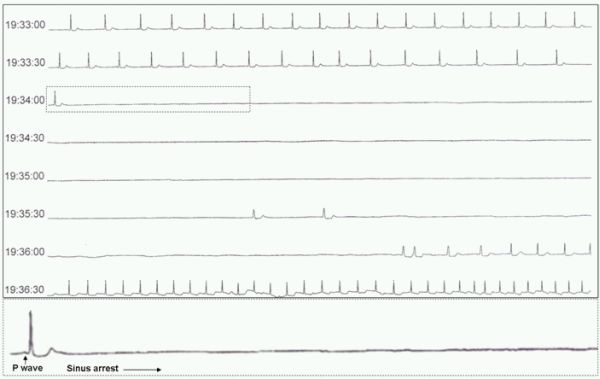
Holter monitoring record demonstrating an extremely prolonged sinus arrest

